# Feeding and the Rest Cure in Typhoid Fever

**Published:** 1904-11

**Authors:** 


### SELECTIONS AND ABSTRACTS.

### Feeding and the Rest Cure in Typhoid Fever.*

In casting about for a subject which would be worthy of your attention this evening I confess that I have had some difficulty in deciding upon the theme which would prove most interesting. On the one hand I might bring before you certain questions which can only be decided by experimental research, and on the other it is possible to call your attention to equally timely, but more practical subjects, which perhaps can be immediately utilized with advantage in practice. In addressing a body of medical men which possesses a wide clinical experience obtained first in the wards of the Presbyterian Hospital, and secondly in private practice, one can not help feeling that he occupies the position of one who comes to obtain information rather than to give it. This is my attitude to-night, and if in the course of my remarks I touch upon subjects which seem to you hackneyed and already exhausted, you must recognize that they are themes which strike me as being of importance in my own experience in which perhaps you may be interested, and from which, in the discussion which I hope is to follow, I may perhaps gain much from the ideas which will be advanced by those who are first courteous enough to listen to me.

The first subject of which I wish to speak is that which deals with the question of the amount of food which should be given to a patient suffering from typhoid fever at the three stages of his disease. I think that every one is agreed that during the first week of his illness the diet should be liquid, since at this time all portions of the body are apparently less able to carry out their functions in a normal manner than they are in the second and third week of the disease. In cases which have considerable severity at onset it has always seemed to me that the digestive system was more disturbed in the first few days of the illness than later on. I

*Read by H. A. Hare, M.D., Professor of Therapeutics in the Jefferson Medical College of Philadelphia, before the alumni of the Presbyterian Hospital, New York, May, 1904.

am one of those whose who are firmly convinced that for many years we have been making a grave mistake in confining patients suffering from typhoid fever to a purely milk diet. This fact was borne in upon me forcibly about seven years ago when suffering from a prolonged attack of typhoid fever, which while it was severe in many respects, particularly in its complications, nevertheless left my brain during most, if not all, of the illness as capable of thought as it is at this moment. While I did not suffer from hunger in the sense of having an appetite, I was continually conscious of the fact that I was underfed, and I also knew that even when in health I could not take large quantities of milk with good results. When it came to the ingestion of considerable amounts of what might be called "hospital milk," I felt that I was receiving little more than water, and many hours of thought convinced me that on the one hand I was being attacked by the bacillus of Eberth, and that on the other my powers of vital resistance were being reduced by partial starvation.

Immediately after this I began giving those cases of typhoid fever which came under my care a varied diet of liquid and semi-solids, and since that time I have been greatly impressed at each term of hospital service by the advantages of this method of feeding. We must recollect that the average adult requires at least 2,500 calories a day for normal existence, and as each quart of milk represents only about 500 to 600 calories, it is evident that for a patient to receive the total number of calories required for the maintenance of his body he must drink not less than four and one-half quarts of milk a day. These facts further impress us with the utter inadequacy of the pure milk diet in this disease. Nor can we help appreciating the fact that we are overloading the organs of absorption and elimination with an excess of fluid, which certainly can not be advantageous, although of course an excess of liquid in typhoid fever is preferable to a lack of it.

Again, I can see nothing in the clinical history or pathology of this disease which in any way justifies us in disordering metabolism by the institution of a rigid single diet; and I doubt if there is a man in my audience who can go to bed for a period of a month or six weeks and receive an absolute milk diet for that period of time without emerging very much the worse for wear.

It is my custom to give all patients after the first week of typhoid from one to two soft-boiled eggs a day in addition to the ordinary allowance of milk, and to vary their diet by the use of curds and whey, rice which has been boiled to a pulp, barley, wheat, and oatmeal gruel, and a cup of corn-starch with vanilla or some other flavoring substance of a like character. As a result I very rarely see the marked ataxia which is so common a symptom in convalescence from typhoid fever. The patient's nutrition is so well preserved that he is but little more emaciated than many cases of acute pneumonia at the time of recovery. Secondary complications like furuncles and bed-sores are unknown, for by the use of a plentiful supply of food the patient's vital resistance is maintained to such a degree that simultaneous collateral infections do not take place. The average case of convalescent typhoid fever is a fair mark for any infection, because it is half-starved.

Recognizing that typhoid fever is characterized by a deficient secretion of digestive juices, all these patients receive hydrochloric acid and pepsin when proteids are administered, and taka-diastase and pancreation when carbohydrates are used. I have never seen any bad symptoms arise as the result of this plan, and I am quite sure that I have seen an immense amount of good follow. I am not friendly to the use of beef tea, which I believe acts as a first-rate culture medium and frequently increases tympanites and diarrhea, the stools becoming fetid under its use.

For many years I have been much dissatisfied with the various theories which have been advanced to explain all the good effects which follow the use of cold bathing in typhoid fever. Most of my auditors will remember that but a few years ago the good effects of cold plunging were supposed to depend chiefly upon the fact that by this means we were able to bring the temperature approximately to normal. Increasing experience has, I think, taught us that the mere reduction of temperature is the least of the results which hydrotherapy brings about. We must believe, on the other hand, that the readjustment of the circulation or the reestablishment of the normal circulatory equilibrium is the chief benefit derived from this method of treatment, and there can be no doubt, I think, that the great increase in the elimination of toxic materials in the urine after the cold bath depends upon this readjustment of the circulation and the increased rapidity with which toxic blood is taken to the kidneys where it can be purified. A number of years ago there appeared in the *Australasian Medical Gazette* a paper by one of the staff of the Brisbane Hospital in which he reported a large series of cases of typhoid fever treated not by the use of the cold bath but by the employment of the tepid bath—a method instituted at first because the climate was so hot that ice was difficult to obtain, and subsequently adhered to because the results which were obtained fully equaled and indeed surpassed those which were reached by the use of ice-cold water. In these baths, as well as in the cold baths of Brand, we all know that active friction is a factor of very great importance, aiding the dissipation of body heat, and the longer I study this matter the more convinced I am that active friction in these cases is an exceedingly important part of the treatment, not only because it aids in the dissipation of heat and readjusts the circulation, but also because it exerts upon the patient beneficial effects which follow the use of massage when we employ the rest cure in the treatment of neurasthenia.

Some months ago, when Dr. Mitchell read his first account of the early cases in which he instituted the rest cure, he took pains to point out that he failed dismally in good results from rest in bed and forced feeding until in addition he provided passive exercise in the form of massage.

Personally I have come to consider that the value of the modern method of treating typhoid fever by cold depends in great part upon the fact that when cold bathing is used, the typhoid fever patient, who is undoubtedly suffering from a form of toxemic neurasthenia, receives throughout his illness a form of rest cure which maintains strength and puts him in first-rate physical condition in much the same manner as we endeavor to do for nervous wrecks in the social world. In other words, I do not believe that the free use of cold water in itself is the chief factor for good in these cases, but that if we amply feed and thoroughly rub our patients, a great portion of the good comes from these measures. In other words, I would advocate the employment of the Weir Mitchell rest cure in the treatment of typhoid fever.

I have also been very much impressed with two additional facts in this connection. One is that equally good results can be obtained in typhoid fever if patients are properly sponged with friction instead of being plunged. The same fall of temperature, the same reaction, the same reddening of the skin without cyanosis and congestion, can be produced by sponging with friction. I have never seen a temperature which could not be reduced by proper sponging, or ice-rubbing, even better than by the plunge bath. The sponging possesses the additional advantage that the patient does not have to be moved from his bed, that the great muscles of the back can receive even more attention than the anterior portion of the body, thereby increasing the dissipation of heat very greatly and preventing creasing of the skin and the formation of bed-sores. For several years I have never plunged my patients, and have always sponged them. My experience is, however, that every new nurse that sponges for me has to be instructed in the proper use of friction, and in any instance in which the temperature fails to fall under this method I feel confident that the fault is in the way the nurse does the sponging and not in the method itself.

The second point is that many patients suffering from typhoid fever are profoundly toxemic when their temperature is considerably below 102½°, the point at which the cold plunge is usually considered wise. Such patients are not given the cold plunge for the very good reason that if they receive it their temperature may become subnormal, and they may collapse. The toxemia is permitted to persist, because the temperature has not risen high enough. Under these circumstances I believe that we should resort to tepid baths. I feel convinced that I have repeatedly saved life by the use of tepid or even hot water used with sponging and friction in toxemic cases which were certainly too feeble to bear cold. By the use of hot or tepid water with friction, circulatory equilibrium can be re-established, toxemia reduced, and even the temperature reduced to normal.

The next point I wish to consider is the question of the administration of alcohol to typhoid fever patients. Before I fed these cases so well I used very much more alcohol than I use at present, and I was firmly convinced of the fact that it did these patients good. I still use it quite largely, but, with good feeding, I can get along with much less than formerly, probably because the patient burns up food products in the body instead of burning up alcohol.

Closely connected with this question of the administration of alcohol is that which deals with the manner in which it can do good in typhoid fever. It has been shown so conclusively by a host of scientific investigators that alcohol is not a true stimulant in the sense in which we employ that term in connection with drugs which increase the functional activity of various organs that we can not ignore the fact that in the past our views concerning its mode of action were erroneous. On the other hand, the experience of a vast number of practitioners has been almost without exception in favor of the proper employment of this drug in typhoid fever, and surgeons innumerable are prepared to assert that iron, alcohol, and quinine are the best means of combating various forms of bacteremia, admitting, it may be, that they know not how they do good, but asserting vehemently that they are invaluable.

At the present time it would seem likely that we are standing on the threshold of therapeutic discoveries which make all previous ones insignificant. Ehrlich's theories in regard to antitoxic bodies, receptors, and haptophores have thrown a flood of light upon many physiological and pathological processes, and I believe are destined to show that many of our therapeutic measures which we have thought rested upon a scientific basis have been purely empirical, in the sense that we have been entirely ignorant of the manner in which their effects have been produced. In other words, as an illustration of this, in the past we have called alcohol a stimulant, thinking only of the circulation, the kidneys, and respiration. It is quite possible that it does not act as a stimulant upon these fnnctions, but upon other functions which are of equal importance in the human body, and are connected with immunity and the ability of our bodies to resist infection. It would seem probable that certain drugs, such as those I have named, for example, may exert an influence upon the body by means of which the processes of artificial immunity are greatly increased. Recognizing the unsatisfactory state of our knowledge in regard to the employment of alcohol in various infections, it occurred to me that this drug might perhaps do good in typhoid and other fevers of an asthenic type associated with bacteremia by increasing the bacteriolytic power of the blood, and I therefore instituted a series of experiments which I reported at the meeting of the Association of American Physicians in Washington in May, 1903, and published in the *Therapeutic Gazette* for May, 1903.

